# Factors Related to Addiction Treatment Motivations; Validity and Reliability of an Instrument 

**Published:** 2016-09-29

**Authors:** Hamid Tavakoli Ghouchani, Shamsedin Niknam, Farkhondeh Aminshokravi, Seyed Kaveh Hojjat

**Affiliations:** ^a^ Department of Health education, Faculty of Medical Sciences, Tarbiat Modares University, Tehran, Iran; ^b^ Addiction and Behavioral Sciences Research Center, North Khorasan University of Medical Sciences, Bojnurd, Iran

**Keywords:** Psychometrics, Motivations, Treatment, Substance Abuse, Outpatients

## Abstract

**Background:** Understanding the motives and reasons for drug treatment is very important. This study aimed to develop a psychometric evaluation to determine the reasons for addiction treatment among outpatients referred to addiction treatment clinics.

**Methods:** This cross-sectional validation study included five phases (i) Item generation (ii) Making an initial questionnaire (iii) Content validity (iv) Reliability analysis and (v) Structure validity. Addiction treatment motivations were identified by reviewing literatures and interviews with 21 stakeholders. A 30-item questionnaire was used for data collection and a random sample of 300 participants completed the questionnaire. The data were analyzed using content validity (CVR &CVI), internal consistency (Chronbach’s alpha coefficient) and exploratory factor analysis (EFA) by SPSS version 16 software.

**Results:** With exploratory factor analysis, 22 items that were remaining jointly explained 60.6% of the variance observed. Inconsistency assessment, Cronbach’s coefficient (α) of items was 0.9. Items with CVIs and CVRs greater than 0.84, remained and factor loading cut off ≥ 0.5 as valid items. They were loaded into four factor solution for the questionnaire, namely: family factors, threats, friend’s factors and self-efficacy.

**Conclusions:** This study suggests a reliable and valid instrument with four factors related to motives of addiction treatment.

## Introduction

“Drug abuse is a chronic, relapsing brain disease identified by compulsive substance seeking and use, despite harmful consequences”^1^. Substance dependency is an illness that can affect anyone, regardless of being male or female, young or old, rich or poor and any race and ethnicity^2^. The prevalence of drug use disorders is estimated 35/1000 persons in the Eastern Mediterranean Region^3^. Addiction is a problem for public health, one of the main causes of crime, disorder, family breakdown and community disintegration^4^ with high costs for both addicts’ population and the society^5^.

Although, different programs for prevention and rehabilitation were designed and implemented, the addicts’ population remained high in most parts of the world^6^. Motivations and readiness for treatment are salient factors^7^. The basic component of quitting addiction is the reason for taking action against addiction^8^. Motivational factors at the beginning of treatment can positively impact success in the treatment^9^.

Social stability; previous experience and expectations of treatment, and higher motivation were predictors of addiction treatment retention^10^. Attitudes towards continued substance abuse, partners and community stigma; perceptions of cessation and drug treatment are significant items for treatment^11^.

Some of the factors that determine addiction treatment include self-hatred, shame and humiliation related to substance abuse, negative beliefs and feelings about addiction, stigma and distrust; positive feeling about acceptance and well-being to life^12^. In the field of treatment, the most significant factors to stop drug abuse proved to be economic, social and empowering individuals^13^.

Addiction treatment intentions are motives ranging from internal to external influences, including a negative impact on oneself and others; influence of family, peers, partners and community stigma ^11^ and similar factors. These are also very important for predicting treatment success. Influence of family, peers and partners are motives behind drug addiction treatment^14^.

Addiction treatment studies have shown that self-efficacy is a major predictor for health behaviors. Motivational level, consequences of addiction and criminal history are other factors to be considered in taking action against addiction^7^. Addiction is a chronic disease; hence, addiction treatment requires long-term management^15^‏. Understanding the role of personal motivation in addiction treatment is very important for a better perception of relapse and treatment retention. There is experimental evidence that treatment motivation and readiness are closely related to retention^16^. Therefore, the factors influencing the addiction abandonment are different.

These are several instruments for measuring factors related to addiction treatment motivations for example: TCU Motivation tests that assess motivation for treatment concerning desire for help, treatment readiness and pressures for treatment^12^, the readiness to change questionnaire in Addiction^17^; Barriers to treatment inventory (BTI) ^18^. But, there is the lack of an instrument for measuring factors related to addiction treatment motivations among outpatient referred to addiction treatment clinics for Iranian conditions.

This study aimed to develop a valid questionnaire to determine the reason for addiction treatment among outpatient referred to addiction treatment clinics, by determining the content validity of measures based on the obtained opinions from specialists and participants, and for evaluating the factor structure of the scale using exploratory factor analysis EFA); and assessing reliability of the questionnaire using internal consistency.

## Methods

This cross-sectional validation study was performed in Bojnourd, North East of Iran from May to September 2014. The inclusion criteria were the addicted people referred to addiction treatment clinics (outpatients) and consent to participate in the study. At least, they used a type of drug. The exclusion criteria included did not agree to participate in the study. Participants were selected using a multistage random sampling method. All participants agreed to complete the questionnaires.

Informed assent and consent were obtained from participants. The study was conducted with approval from Tarbiat Modares University’ Institutional Review Board and Ethical Committee.

**Figure 1:**
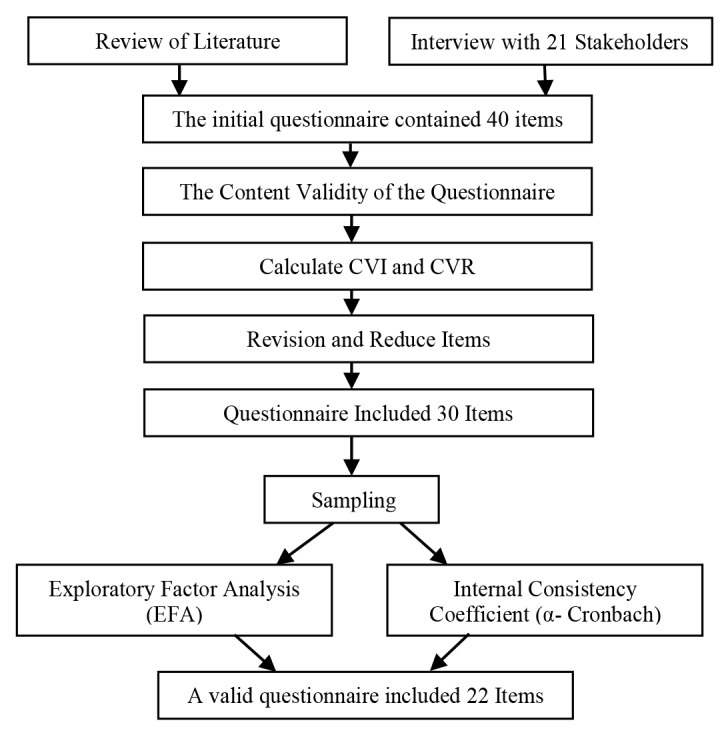
A Flow Chart Depicting the Process Used to Evaluating the Psychometric Properties

Data collection methods were based on anonymous questionnaires completed by the participants, and also among the illiterate people by trained psychologists in ten clinics. Patients completed a questionnaire on a Likert scale of 1-5, strongly disagree= 1, disagree= 2, no idea=3, agree= 4 and strongly agree= 5. The questionnaire was developed through the following steps (Figure 1):‏

### I. Item generation

Interview and review of literature identified reasons and motivations associated with the action and continuity of abandonment

### A. Interview with 21 participants.

Participants were volunteers, including physicians, psychotherapists (with working experience in addiction treatment clinics) and outpatients. The main question was “What are the reasons for treatment retention among clients in addiction clinics”. Briefly, the following steps were taken for conventional content analysis:

- Writing and implementation of the interview
- Reading the text for understanding
- Determining the meaning of primary codes
- Classification of the same primary codes in categories
- Determining the content within the data.

The interviews lasted for 30-40 min. All interviews were recorded and transcribed verbatim; of course, the verbal permission had already been taken for recording and transcription.

The consistency of ideas and experiences was examined in the transcripts. Then, a detailed list of meaning units was formed from each interview transcript. They were coded into the various sub-categories. The categories were formed based on the similarities and differences between each sub-category.

In addition, the meaning units and sub-categories were reviewed and approved by some of the participants and experts in the field of qualitative research and addiction treatment.

### B. Review of literature

One hundred and twenty cross-sectional studies published by Elsevier, Science Direct, external and internal valid scientific sites (mostly specific and related to addiction) were chosen by searching in Google Scholar. Key words used were “addiction, treatment, motivation, readiness, maintenance and factors”. Finally 23 articles (13 internal and 10 external) were used and evaluated. They have greater sample size and more citations than the others.

These items were associated with the action and continuity of abandonment. For example, negative attitudes towards consumption, external pressures, the consequences of abuse, fear of legal troubles, humiliation, loss of job, the family's insistence, for children and parents, saving family communications, family support (family’s care and company, assistance of spouse) and others.

### II. Making an initial questionnaire

Based on literature review and interviews, a framework was identified in order to develop the initial questionnaire. The initial questionnaire contained 40 items. The content validity of the questionnaire was examined by thirteen specialists from different disciplines, including health educator, physicians and psychotherapists. The purpose of this step was to ensure that the instrument was clear and culturally relevant.

### III. Content validity

Content validity was applied in two phases (qualitative and quantitative). The qualitative phase was conducted by 13 experts who reviewed the items of the questionnaire for grammar, wording, item allocation and scaling. The quantitative phase was conducted to calculate CVI and CVR. CVR examines the essentiality of each item for the Iranian culture by using 3-points rating scale (essential, useful but not essential and not essential). The CVR for every item was calculated using the formula CVR = [Ne − (𝑁/2)] ÷ (𝑁/2) (Ne is the number of panelists indicating "essential" for each particular item and N is the total number of panelists). The numeric value of CVR was determined by Lawshe table, accordingly, an acceptable CVR value for 13 panelists is 0.54(19). To obtain CVI for relevancy, simplicity and clarity of each item, ordinal scale with four possible responses were used. The responses included a rating from 1 = not relevant, not simple and not clear to 4 = very relevant, very simple and very clear. The number of those judging the item as relevant or clear (rating 3 or 4) was divided by the number of content experts. Polite and Beck recommended 0.79 as the acceptable lower limit for CVI value 20).

Consequently, 10 items were removed and a primary version of the questionnaire with 30 items was developed (CVI >0.79 and CVR >0.54).

### IV. Exploratory factor analysis

Construct validity was determined through exploratory factor analysis (EFA). EFA was performed to determine the dimensionality of the questionnaire using the principal component analysis with varimax rotation. Factor loading values of 0.5 or higher were considered acceptable and showed that there was an important relationship between items and factors. In order to evaluate sampling adequacy to perform a satisfactory factor analysis, KMO Measure of Sampling Adequacy and Bartlett test was high values of KMO (more than 0.7) generally indicated that a factor analysis may be useful with the data. The criteria used to determine the subscales (factors) were minimum Eigenvalues >1.00 (Kaiser Criterion) ^21^.

### V. Reliability

To determine the reliability of the instrument, the internal consistency was tested using the Cronbach’s alpha coefficient. Reliability of the scale was determined by computing Cronbach’s Alpha as an internal consistency coefficient (α>0.7). Cronbach’s coefficient alpha (α) was calculated separately for total scale and each item.

At the first stage, sampling was conducted based on the cluster method. Each cluster was in different sections of the city. Ten addiction treatment clinics were chosen, among one hundred and four clinics within the designated metropolitan area of the study population. Each clinic was in different section of the city at the second stage, patients in each clinic were selected through simple sampling method, based on performance capacity of the data collection.

The sample size was estimated based on the number of items in the questionnaire multiplied by 6-10 as recommended (300 participants). The sample size was determined by scientific references in exploratory factor analysis^19^. Data were analyzed using SPSS 16 software (Chicago, IL, USA).

## Results

A total of 300 participants, 80.6% male and 19.4% female completed the questionnaire. The respondents were aged between 16 and 71 year of mean age of 39.4±12.06. Most of them were married (78.62% married, 14% single, and 7.38 % divorced). They used opium (39%), cooked dross (36%), heroin (5.7%), methamphetamine (10%) and others were multiple drug user. The average lifetime drug use among participants was 15.12±10.03 year (range = 1 to 46 year).

Content validity was calculated. According to the Lawshe table Items with CVI >0.79 and CVR >0.54 was remained. Construct validity was determined. In the first step, Kaiser-Meyer-Olkin (KMO = 0.88) and Bartlett's Test (*P*<0.01, df= 595 , x2=5195.65 ) showed the adequacy of the sample size. Principal component analysis with Varimax rotation identified eight factors (Eigenvalues >1.0, factor loading cut off ≥ 0.5) which explained 60.6% of the variance in the data.

Next, 8 items were removed from the questionnaire that seemed to be similar or unrelated items. The remaining 22 items were subjected to principal components analysis with varimax rotation that showed a good fit of 4-factor solution for the questionnaire.

The four factors were: Family’s Factors (five items), Treats (eight items), Friend’s Factors (four items), and Self-Efficacy (five items) and explained variance (%) of each factor (Table 1).

**Table 1:** Factor Loadings of addiction treatment motivations obtained and Variance Explained in exploratory factor analysis (EFA)

**Items**	**Factor loadings**	**Variance in EFA**
**Family Factors**		15.54
1	I want treatment because my family encourages me for treatment	0.808	
2	My family support me for continuing treatment	0.765	
3	My family is insisting for my treatment	0.750	
4	If I'm successful in treatment, it would be a big relief for my family	0.745	
5	My family is suffering because of my addiction	0.734	
**Threats**		
1	I want treatment because my relationship with my partner at risk/not able to marry	0.824	16.91
2	It is possible, I catch to mental or physical diseases	0.675	
3	I might lose my job/not able to get a job	0.671	
4	My honor might be compromised	0.664	
5	I might be abandoned by my family	0.640	
6	‏notoriuos became I and ‏appearance my changed has adddicton	0.627	
7	I will be suffering from physical and mental health disorders	0.564	
8	My addiction might cause many family issues	0.527	
**Self-Efficacy**		12.81
1	I am well prepared for treatment	0.697	
2	I am able to follow up my treatment	0.692	
3	I can that confident I'm stopping using drugs	0.659	
4	I see the people who are successful in treatment. I am more determined in the treatment	0.654	
5	I can control my relationship with my friends who are using drugs	0.634	
**Friends Factors**		15.33
1	My friends are insisting for my treatment	0.838	
2	I have friends I can trust them and talk about my treatment issues.	0.815	
3	My friends and relatives encourage me for treatment	0.802	
4	My friends and colleagues trust me more	0.761	

Internal consistency of the questionnaire was examined by computing the Cronbach’s alpha that gave a satisfactory value of 0.896. Cronbach’s coefficient alpha (α) was calculated separately for total scale and each item (Table 2).

**Table 2:** The Results of Reliability Obtained from Chronbach’s Alpha Coefficient

**Items**	**Chronbach’s Alpha Coefficient**
**Family factors**
1	0.86
2	0.88
3	0.87
4	0.86
5	0.86
Total	0.89
**Threats**
1	0.82
2	0.81
3	0.82
4	0.79
5	0.81
6	0.81
7	0.82
8	0.81
Total	0.83
**Self-efficacy**
1	0.70
2	0.69
3	0.66
4	0.65
5	0.63
Total	0.82
**Friends factors**
1	0.86
2	0.85
3	0.85
4	0.87
Total	0.89
Overall	0.89

## Discussion

According to the results, four factors were related to motives of addiction treatment including family factors, threats, friend’s factors and self-efficacy, which is in line with previous studies^7,14,16^.

The family and friends factors are the two components related to addiction treatment. Family and friends factors included supported by them and motivation to comply with them. In this regard, the likelihood of drug abuse was greater among those who engaged in emotional and social problems, such as psychological problems and family dispute compared with their counterparts who did not engage in such problems^22^.

Family support is a positive factor in addiction rehabilitation^23^. Family and friends support is a type of emotional support^24^. Family support and other types of social support are mechanisms of changes in treatment ^25^. Social support is one of the essential services to stop or reduce substance abuse. In other words, motivation to comply with family and friends were associated with the action and continuity of addiction treatment ^26-27^.

This study showed that the role of family and friends is common in social support and motivation to comply among the addicted population. Previous researches showed some similarities with our results^14,23^.

The present study indicated that “threats” was another significant factor in addiction treatment. Threats vary and include loosing job and money, the consequences of abuse, fear of legal troubles, going to jail, losing families; and the severity^28^. Treatment motivation was positively correlated with problem severity^29^ and the consequences of drug abuse were important predictors of motivation to addiction treatment^30^.

The other factor is self-efficacy; in this study, it was measured using five items. According to Bandura self-efficacy is the most important precondition for behavioral change ^31^, self-efficacy is a psychological construct of central importance in understanding human behavior ^32^ and directly affects on performance ^33^. It is one of the directly related predictors in quitting ^34^. Increasing the self-efficacy is the most effective in substance abuse treatment ^32,35^. It may be the best effective addiction treatment that increases self-efficacy^36^. Higher self-efficacy, is a predictor of making a quit attempt^37^. Self-efficacy is as an important predictor of outcome, or as a mediator of substance abuse treatment ^38^.

The major limitation of this study was the lack of control on drug types used by the participants. Confirmatory factor analysis was another limitation because it needed new samples and more time.

## Conclusions

This study designed questionnaire with 22 items and suggested a reliable and valid instrument with four factors related to motives of addiction treatment, including: family factors, threats, friend’s factors and self-efficacy. The questioner can be used as an instrument in substance abuse treatment because it is valid and reliable.

## Acknowledgments

We would like to appreciate all the participants and participating clinics and others who helped us in this research.

This manuscript was based on the thesis of Hamid Tavakoli Ghouchani, with reference number 52/4470 D, supported by the Research and Technology Deputy of Research and Technology (Tarbiat Modares University).

## Conflict of interest statement

The authors declare that there is no conflict of interest regarding the publication of this paper.

## Highlights

- Family factors, threats, friend’s factors and self-efficacy are significant factors in substance abuse treatment.
- There is the lack of an instrument for measuring factors related to addiction treatment for Iranian conditions.
- This study suggests a reliable and valid questionnaire to determine the reason for addiction treatment.
